# Operative implications of the small aortic root

**DOI:** 10.1186/1753-6561-6-S4-P4

**Published:** 2012-07-09

**Authors:** LM Lucuta, SL Mosteoru, M Gaspar, H Feier

**Affiliations:** 1 “Victor Babeş” University of Medicine and Pharmacy, Timişoara, Romania; 2 Timişoara Institute of Cardiovascular Medicine, IInd Cardiovascular Surgery Department, Romania

## Introduction

Replacement of the aortic valve with a mechanical prosthesis when the annular diameter is small, meaning 21 mm or less, can present significant hemodynamic and technical problems, including patient-prosthesis mismatch and persistent heart failure. The aim of this paper is to assess the operative implications of the small aortic root during surgery.

## Methods

Our study comprises of 46 patients operated during the last 3 years in the IInd Cardiovascular Surgery Department who received 19 and 21 mm aortic valves. There were 20 males (43.48%) and 26 females (56.52%) in our cohort. The mean age was 57 years (ranging from 17 to 78). The most frequent diagnosis was degenerative aortic stenosis (59%) followed by rheumatic aortic disease (22%). The preoperative indexed aortic valve orifice was 0.58 cm^2^/m^2^ and the mean maximum transaortic gradient was 82 mm Hg. The follow-up period was around 22.58 ± 12.19 months.

## Results

There were 3 deaths out of which 2 were intraoperative in our series (6.52% and 4.35%, respectively). All had 19 mm valves implanted, of which one after aortic annulus enlargement and their theoretical indexed effective orifice area was smaller than 0.65 cm^2^/m^2^ in all cases. A total of 8 (17.39%) patients had 19 mm valves and 38 (82.61%) had 21 mm prosthetic valves.

## Conclusions

The small aortic root is a serious operative risk. Based on the available valve types and preoperative body surface area the minimal recommended prosthesis size can be determined. Enlargement of the aortic annulus should be considered more frequently in these cases.

